# Social Environment and Mental and Behavioral Health Outcomes in Older Adults: A Critical Review

**DOI:** 10.1093/geroni/igaa057.1512

**Published:** 2020-12-16

**Authors:** Atami Sagna De Main, Bo Xie

**Affiliations:** The University of Texas at Austin, Austin, Texas, United States

## Abstract

Social environments are complex and critical to older adults’ health. Older adults are vulnerable to lack of social connectedness, social engagement and social contribution. 10-43% of community-dwelling older adults suffer from social isolation and loneliness in the United States. Despite the growing evidence on the impact of social environment on older adults’ health, it remains that the information about older adults’ social environment and its relationships to mental/behavioral health is fragmented. The purpose of this study is to determine the state of the science on social environment components (social connectedness, social engagement, social contribution) and mental/behavioral health (depressive symptoms, anxiety, psychological well-being, substance use, mental healthcare utilization) among community-dwelling older adults (65+ years). Five databases - CINAHL, PubMed, PsycINFO, PsycARTICLES, SocINDEX - were systematically searched using PRISMA guidelines to identify relevant articles from 1990 to 2019. Eleven articles are included in this review, illustrating relationships between social environment and mental/behavioral health. The studies found that poor social connectedness, social engagement, and social contribution were significantly associated with older age, poor perceived health, depression and anxiety symptoms, poor psychological well-being, hopelessness, having multiple chronic conditions, and functional limitations. Low social connectedness was significantly related to poor utilization of mental health services. No significant association was found between social environment and substance use in older adults. The findings of this review add to the literature related to social environment being relevant to older adults’ mental/behavioral health and highlight the need to further our understanding of their dynamic relationships and changes over time.

